# Role of Sodium in the RprY-Dependent Stress Response in *Porphyromonas gingivalis*

**DOI:** 10.1371/journal.pone.0063180

**Published:** 2013-05-06

**Authors:** Karthik Krishnan, Margaret J. Duncan

**Affiliations:** 1 Department of Microbiology, The Forsyth Institute, Cambridge, Massachusetts, United States of America; 2 Department of Oral Medicine, Infection and Immunity, Harvard School of Dental Medicine, Boston, Massachusetts, United States of America; Centre National de la Recherche Scientifique, Aix-Marseille Université, France

## Abstract

*Porphyromonas gingivalis* is a Gram-negative oral anaerobe which is strongly associated with periodontal disease. Environmental changes in the gingival sulcus trigger the growth of *P. gingivalis* and a concurrent shift from periodontal health to disease. Bacteria adjust their physiology in response to environmental changes and gene regulation by two-component phospho-relay systems is one mechanism by which such adjustments are effected. In *P. gingivalis* RprY is an orphan response regulator and previously we showed that the RprY regulon included genes associated with oxidative stress and sodium metabolism. The goals of the present study were to identify environmental signals that induce *rprY* and clarify the role of the regulator in the stress response. In *Escherichia coli* an RprY-LacZ fusion protein was induced in sodium- depleted medium and a *P. gingivalis rprY* mutant was unable to grow in similar medium. By several approaches we established that sodium depletion induced up-regulation of genes involved in oxidative stress. In addition, we demonstrated that RprY interacted directly with the promoters of several molecular chaperones. Further, both genetic and transcription data suggest that the regulator acts as a repressor. We conclude that RprY is one of the regulators that controls stress responses in *P. gingivalis*, possibly by acting as a repressor since an *rprY* mutant showed a superstress reponse in sodium-depleted medium which we propose inhibited growth.

## Introduction

Periodontitis is a chronic inflammatory disease induced by polymicrobial infection of the periodontium. If untreated, the infection can lead to tooth destabilization due to destruction of supporting tissues. *Porphyromonas gingivalis* is a gram negative, commensal, black-pigmented, anaerobic oral bacterium and is widely considered as one of the causative agents of periodontitis due to its strong association with the disease [1]. Accordingly, this organism is studied extensively and several factors that contribute to its pathogenicity have been characterized [2]. *P. gingivalis* is part of the commensal microflora of the supra- and subgingival plaque biofilm [3] which can colonize gingival epithelium without apparent disease activity [4], [5]. The shift from periodontal health to a pathogenic state may partly depend on changes in the gingival microenvironment caused by early colonizing bacteria [6], [7], as well as host conditions including changes in ion concentration, exposure to blood, saliva, and host immunological factors.

Bacterial cells perceive and respond to environmental stimuli in order to ensure their survival. One type of response mechanism is mediated by two-component signal transduction in which a sensor, a transmembrane histidine kinase, perceives a change in the cell environment, and autoactivates by phosphorylation. The activation signal is relayed by phospho-transfer to activate the cognate response regulator, a DNA-binding protein that regulates transcription of response-specific genes. The RprY response regulator of *P. gingivalis* is the focus of the present study. RprY is an “orphan” in that the usual close genetic linkage to a candidate cognate kinase gene is absent, and hence, neither cognate kinase nor activating signals are yet known. However, in Bacteroidetes and other oral pathogens, the *rprY* homolog is found together with its cognate kinase gene *rprX*, and when the *rprXY* two component system (TCS) from *B. fragilis* was expressed in *E. coli* it conferred low level tetracycline resistance that correlated with a decrease in *ompF* expression suggesting that *rprY* may belong to EnvZ-OmpR family of transcriptional regulators [8].

Our previous investigation showed that in *P. gingivalis* several genes associated with oxidative stress and sodium translocation were part of the RprY regulon, suggesting a possible role in Na**^+^** metabolism and the response to redox changes [9]. The goals of the present study were to determine the activation signal(s) for *rprY* and clarify its role in the oxidative stress response. We found that the regulator was essential for growth of *P. gingivalis* under sodium-limited growth conditions. While the parent strain adapted and was able to grow, albeit slowly, under these conditions, an *rprY* mutant strain could not, indicating that RprY was essential for the response to this stress and hence viability. By transcription profiling and metabolite analyses we determined that sodium limitation induced an oxidative stress response in both parent and *rprY* mutants strains. However, the response was highly amplified in the mutant and was accompanied by dysregulation of genes encoding protein chaperones. We found that RprY interacted directly with the promoters of several chaperone genes, and collectively our data indicate that the regulator acts as a repressor of their expression. We hypothesize that in the absence of RprY, *P. gingivalis* is unable control oxidative stress and protein chaperone responses compromising growth of the mutant under stress conditions.

## Materials and Methods

### Strains, Media and Growth Conditions

*Escherichia coli* strains used in this study were derivatives of *E. coli* K12 (Table 1). *E. coli* was grown and maintained in Luria-Bertani (LB) media supplemented with ampicillin (100 µg/l) as required. For RprY-LacZ expression assays cultures were grown at 37°C with aeration in modified LB broth. Briefly, the medium was reconstituted from individual components (tryptone and yeast extract). Sodium chloride (NaCl; 171 mM) was either included (LB) or omitted (LB0). To investigate the role of ion or stabilizers on RprY-LacZ expression, NaCl (171 mM), KCl (171 mM) or sorbitol (500 mM) were added to NaCl –depleted LB (LB0).

**Table 1:** Primers, strains and plasmids used in this study.

Primers	Sequence 5'- 3' forward/reverse (amplicon size in bp)	Reference
***rprY-lacZ*** ** cloning**		
*rprY*	ACCCGGGGATTTTTATTCCTGCCATAAC/GGGATCCTTACTATCGTCCTCGCAGAG (821)	This study
***rprY*** ** cloning in pET28a**		
*his-rprY*	AAGCTTGCATGCCTGCTCAGACCTCTTTGATCAACTC/	This Study
	TATACATATGGAAGAAAAAACAAGAATCTTTCTCT	
***rprY*** ** cloning in pFD288**		
rprYHis3	TAGACGTCTCAGTGGTGGTGGTGGTGGTGGACCTCTTTGATCAACTCCTCA	This study
KAR6a	ACCCGGG GAT TTT TATT CCT GCC ATA AC	This study
**OxyR deletion**		
3-way-oxyRF1	CGAGTGGCGATGAGCGTCAGGCC	This study
3-way-oxyRR2	TTCCGAGCTTTCTTCTTTTGCCGT	This study
3-way-oxyR R1-EM	TGTAGATAAATTATTAGGTATACTACTGACAGCTTCGGGAAGGATCGTTTTGTATTAG	This study
3-way-oxyR F1-EM	ACCGATGAGCAAAAAAGCAATAGCGGAAGCTATCGGACGAATCATATTTCCTATTTTTG	This study
**RprY deletion**		
KK108	GAGATAGAAGCATTAGAACTAATGGGCTACTAAAGGGCT	This study
KK109	GGAGATAATTCGTTGTTTATCCAGCTGAGAACGGAATGAG	This study
KK111	CTCATTCCGTTCTCAGCTGGATAAACAACGAATTATCTCC	This study
KK112	AGCCCTTTAGTAGCCCATTAGTTCTAATGCTTCTATCTC	This study
KK107	CTCGTGTTTATGGTTCCCAA	This study
KK110	GTCAGGGGAGGCTCTGCTTC	This study
**qPCR**		
16S	TGTTACAATGGGAGGGACAAAGGG/TTACTAGCGAATCCAGCTTCACGG(118)	[42]
*sod*	AAAGAGCGAAGGCGGTATCT/CGAATGAGCCGAATTGTTTGTC(140)	[42]
*ahpC*	TCAAACTCAATGCCTATCACAATG/GATAGAAAACGACCAAAGACCACT(87)	[42]
*rprY*	CCATCGCGATCGATGATCAGGTAA/GGCATAGTTGCGTTCAAGGGTTTC(104)	[42]
PG0275	ATCGGCAGATTGCCCATCGT/ATGCCGGTAAACTCACCGTCT(122)	This study
PG1286	CAAGGCCGAAATGTGGTCTTCA/TCCATCATATCGTAGGCGTGCT(134)	This study
PG1134	CTCATCATCGGTTCCGGACCT/TTCCACCTCGGTCGTAGTCGT(125)	This study
PG2008	GGATCCGTTACAGGTACCGTAGT/ATTACCCGAAGGGATTCCCTTGA(141)	This study
PG0209	GGTGAGCACCACAAGAATCAAG/GCCGGAGCATACATTGCTATC(144)	This study
PG1321	GAGGTGGCTATGCACAGGTAC/GTCGCAAGTATTGCGGTTCTG(147)	This study
*groES*	CCTCTCAAGGGTGAAGTAATCGCT/ATTTGCCGTAGAGTACGGTGTCTC(91)	[42]
*groEL*	CGGCTACATCTCTCCCTACTTCGT/GAGGATCGGGAGCATCTCTTTCAG(121)	[42]
*clpB*	CTGCTCAAGGCCGTTATGGATCA/CTGGTTGGTCTCATGCGACAGA(160)	[42]
*dnaK*	CTGACCGGTGAGGTAAAGGATGTC/CTTCGTCGGGATAGTGGTATTGGC(120)	[42]
*htpG*	CGGTCGCTGACCGTGATCGT/TCTCCAATGCCGTGGATGCT(132)	[42]
**EMSA probes**		
*groES*	CACCTTTTTTCCGACCGACT/TGTTGCTTGGTTTGTTATTGTTAGT(215)	This study
*dnak*	TGTACAGAGTCATTATTCGT/TCCCATAATTCTATATTCGT(199)	This study
*htpG*	AATAAAAATTTTCTCAGACCACGT/CATAGTGTATTATTATCTGTTAATGT(203)	This study
*clpB*	GTTCTGAGTTTCATAGTAGGGTA/GCTGTGTTTGTTGTTACCAGA(229)	This study
*sod*	GTTCGTGAGTCATAACGTCT/TTCGTCATGGAATGAATCGGT(164)	This study
*ahpC*	ACTGCGCCTTCTTAATGTTT/AGTATGAACGTTTGTTTCAGG(356)	[9]
**Strains and plasmids**		
**Name**	**Description or Genotype**	**Reference**
*P. gingivalis*	ATCC33277(type strain)	Coykendall et al
*oxyR* mutant	ATCC33277ΔoxyR::*ermF-ermAM*	This study
*rprY* mutant	ATCC33277 *rprY::ermF-ermAM*	[9]
*rprY* tet deletion	ATCC33277 Δ*rprY::tetQ*	This study
*E. coli* DH5a	*fhuA2* Δ*(argF-lacZ)U169 phoA glnV44 Φ80* Δ*(lacZ)M15 gyrA96 recA1 relA1 endA1 thi-1 hsdR17*	Invitrogen
*E. coli* TB28	*rph-1 ilvG rfb-50 lacZYA <frt >*	[43]
pRS414	lacZ protein fusion vector, *lacZYA* lacks promoter, RBS and translational start, apr, colE1 origin	[44]
pFD972a	Expression vector, 9.1 kb; tetQ; (Apr) Tcr	[45]
pRprylacZ	pFD972a *rprY-lacZ*	This study
pFD288	Bacteriodetes shuttle vector	[15]
pFDrprY	pFD288*rprY*	This study

*P. gingivalis* ATCC 33277 and erythromycin-resistant (Erm^R^) *rprY* and Δ*oxyR* isogenic mutants were maintained on tryptic soy agar (TSA) supplemented with 5% sheep blood, menadione (1 µg/l), hemin (1 µg/l) and, when appropriate, erythromycin (5 µg/ml). Strains were grown in an anaerobic atmosphere containing 85% nitrogen, 5% hydrogen, 10% carbon dioxide either in sealed bags (Anaeropaks, Mitsubishi) or an anaerobic chamber (Coy). Trypticase Soy broth (TSB) was used for NaCl- depletion experiments with the medium constituted from individual components (pancreatic digest of casein, papaic digest of soybean, dextrose) with or without NaCl (85 mM), i.e. normal and NaCl-depleted TSB.

### Plasmid Construction and RprY Recombinant Protein Purification

PCR primers used for strain constructions are listed in Table 1. Fusion plasmid pFD*rprY- lacZ* was constructed in a three- step procedure. The *lacZ* gene fragment from pRS414, an *E. coli lacZ* protein fusion vector, was PCR amplified and cloned into plasmid pJET (Fermentas). The fragment lacks a promoter and translational start codon which was provided by cloning a DNA fragment containing 776 bp DNA upstream and 41 bp downstream from the translational start of *P. gingivalis rprY* creating the protein fusion. The complete *rprY-lacZ* hybrid fragment was then cloned into pFD972a [10].

For recombinant RprY protein purification the *rprY* gene of *P. gingivalis* ATCC 33277 was PCR amplified using primers listed in table 1 and cloned into Nde1 and HindIII digested pET28a expression vector (Novagen). The resulting plasmid was transformed into *E. coli* BL21 DE3 (Novagen) for expression of the N-terminal 6X histidine tagged RprY recombinant protein which was purified using an Ni^+2^-NTA agarose affinity purification column. The eluate was dialysed and further concentrated by treating the membrane with polyethylene glycol (Sigma). The final protein concentration was determined using a Nanodrop 8000 (Thermo Scientific).

### β-Galactosidase Assays

A Pierce yeast β -galactosidase assay kit was used according to the manufacturer’s instruction. Briefly, an overnight culture of the *rprY-lacZ*- containing *E. coli* strain was diluted 1∶50 in LB broth with and without added NaCl and grown for 2 hrs with shaking at 37°C. An aliquot of culture (350 µl) was added to a lysis and assay solution mix (350 µl), vortexed and incubated until sufficient color had developed. The assay was stopped by addition of 300 µl of assay stop solution. Cell debris was removed by centrifugation and the color was measured in spectrophotometer at OD_420 nm_. Miller units were calculated as described previously [11].

### Construction of *oxyR* Deletion and *rprY-*complemented Mutant Strains

Three- way Soeing PCR [12] was performed to fuse 500 bp upstream and downstream sequences of the *oxyR* ORF to *ermF-ermAM* cassette [13]. The fused fragment was cloned into cloning vector pC2.1 TA (Invitrogen) which was electroporated into *P. gingivalis* strain ATCC 33277. Transformants were selected for erythromycin resistance generated by a double cross-over event which was confirmed by PCR.

Similarly, 552 bp upstream and 463 bp downstream sequence of *rprY* was fused with *tetQ* cassette [14] by Three-way Soeing PCR. The resultant PCR fragment was electroporated into *P. gingivalis* strain ATCC 33277 and transformants were selected for tetracycline resistance (2 µg/ml). A 1547 bp fragment containing the complete *rprY* ORF, 777 bp upstream sequence and restriction site linker was amplified from the ATCC 33277 genome. The fragment was digested with appropriate restriction enzymes and ligated to the similarly treated pFD288 plasmid vector [15]. The final plasmid, pFD*rprY*, was electroporated into ATCC 33277 Δ*rprY*(tetQ) strain and ErmR transformants were selected.

### *Porphyromonas gingivalis* Growth and RNA Isolation for Microarray and qRT-PCR Analyses

Parent ATCC 33277 and *rprY* mutant strains were grown in tryptic soy broth to exponential growth phase (OD_550_ 0.4–0.6). Cultures were split into two equal parts, centrifuged, and cells resuspended in pre-reduced modified TSBH broth with or without NaCl. Cultures were incubated further for 2 hr anaerobically, centrifuged and RNA either isolated immediately or the cell pellet was resuspended in RNA Later (Ambion) for storage. A control experiment was performed to rule out an oxidative stress response due to brief handling of cells outside the anaerobic chamber, i.e. the parent strain was grown in complete TSB medium and prepared as above. RNA was isolated immediately from one of the centrifuged cell pellets. The cells from the other pellet were resuspended in pre-warmed and pre-reduced complete TSB medium and incubated for 2 hr, 37°C in an anaerobic chamber, then centrifuged and RNA was isolated.

In all experiments RNA was isolated using an Epicenter kit according to the manufacturer’s instructions. Contaminating DNA was eliminated by treatment with Turbo DNase (Ambion). Isolated RNA was monitored for DNA contamination by PCR and RNA integrity was visually assessed after gel electrophoresis. The concentration and purity of RNA was determined using a Nanodrop1000 or 8000 Spectrophotometer (Fisher Scientific).

### Microarray Analysis

Microarray slides were fabricated based on annotated open reading frames in the genome of strain W83, therefore gene numbers used in this work are based on strain W83 annotation in order to avoid confusion. The slides were provided by the J. Craig Venter Institute and their in-house protocol was used for cDNA synthesis and microarray processing. Briefly, 5–15 µg RNA was reverse transcribed using Superscript III (Invitrogen) or Smartscribe (Clonetech) reverse transcriptase, random hexamers (Invitrogen 3 µg/ml), RNase Out (Invitrogen) and 25 mM dNTP-aaUTP mix. Synthesis was carried out for 16–20 hr at 42°C and RNA was hydrolysed using 0.5 M EDTA and 1 M sodium hydroxide followed by incubation at 65°C,15 min, followed by addition of Tris (1 M, pH 7.4) to neutralize the pH. The cDNA was purified (Qiagen Qiaquick PCR purification kit) then precipitated by sodium acetate -ethanol treatment and resuspended in 4.5 µl of 0.1 M sodium carbonate buffer (pH 9.5) and 4.5 µl of Cy3 or Cy5 dyes (Amersham Biosciences). cDNA labeling reactions were carried out for at least 2 hr in the dark and quenched with 100 mM sodium acetate (pH 5.2). Uncoupled dye was removed (Qiaquick PCR purification kit) and labeled cDNAs were mixed together and precipitated by sodium acetate- ethanol treatment. Labeled cDNA was hybridized onto the microarray slides for 16 hr, 42°C. Washed slides were scanned in a Genepix 4000B (Axon) scanner and the spot intensities measured with Genepix 6.0 software. The data were analysed using Significant Analysis for Oral Pathogen Microarray Data (SAOPMD) tools available at the Bioinformatics Resource for Oral Pathogen (BROP) website (www.brop.org) hosted by The Forsyth Institute. The combined data was obtained by analysis of all repeats within and between arrays using LIMMA (Linear Models for Microarray package; http://bioinf.wehi.edu.au/limma/; [16], [17]).

### Quantitatve RT- PCR Analysis

Quantitative RT-PCR was used to quantify relative-fold changes of differential expression and to validate microarray data. iQ SYBR Green super mix (Bio-Rad) was used according to the manufacturer’s instructions. RNA (1 µg) was reverse transcribed with a RevertAid First strand cDNA synthesis kit (Fermentas) according to the instructions provided. Real-time PCR quantitation of double-stranded DNA fragments was performed with an iCylcer (BioRad). A three-step protocol was used for PCR amplification: 95°C, 3 mins for initial denaturation, 50 cycles at 95°C for 15 s, 55°C for 15 s and 72°C for 15 s. A melt curve analysis was performed to determine the generation of a single amplicon under the following conditions: 95°C for 1 min, 55°C for 1 min, and 55°C to 95°C with a heating rate of 0.5°C per 10 s. Each experiment was performed in triplicate and mean values of at least two independent experiments is shown. Fold- changes in gene expression between the samples were determined as previously described [18].

### Electrophoretic Mobility Shift Assay (EMSA)

The intergenic region upstream of the start codon of the gene of interest was PCR amplified using Takara polymerase (Clontech, Takara Bio) or platinum PCR supermix (Invitrogen) and primers listed in the table 1. The DNA fragment was resolved on a 1% agarose gel and purified using a QIAquick gel extraction kit (Qiagen). Digoxygenin (DIG) labeled probes were prepared using DIG labeling Kit (Roche) according to the manufactures instructions. Electrophoretic mobility assays using recombinant RprY were performed as previously described [9], with the addition of poly[d(I-C)] (0.01 ug/ul) to the binding buffer.

### Metabolomic Analysis

Mid-logarithmic phase (OD_550_ 0.4–0.6) cultures of *P. gingivalis* parent and *rprY* mutant strains were split into two and cells were pelleted by centrifugation. Pellets were resuspended in either complete or NaCl-depleted TSB. Media used for resuspension were pre-warmed and reduced in an anaerobic chamber overnight. Resuspended pellets were incubated anaerobically for 2 hr, 37°C, then centrifuged to pellet the differentially- treated cells. Cell pellets were flash frozen in an ethanol-dry ice bath and stored at −80°C. The samples were shipped to Metabolon Inc. (NC) on dry ice for metabolomic analysis.

Samples were analysed by GC/MS and LC/MS/M as previously described [19], [20]. Identification of metabolites was performed by automated comparison of the ion features with reference library of chemical standards developed at Metabolon Inc. Welch’s two-sample t-tests were performed for pair-wise comparison of NaCl-depleted and normal samples also between two different genotypes (parent vs *rprY* mutant). Two- way Anova was used to identify biochemicals exhibiting a significant genotype effect, treatment effect (NaCl presence or absence) and genotype-treatment interaction.

## Results

### Sodium Depletion Induces Expression of the *P. gingivalis rprY* Promoter-LacZ Fusion in *E. coli*

**Figure 1:**
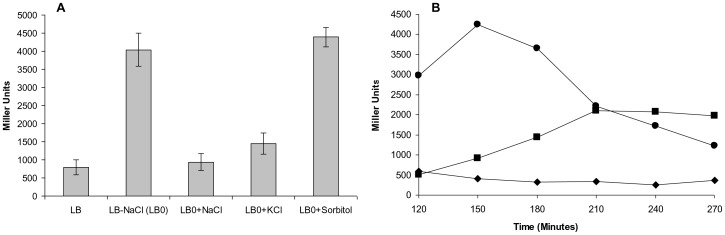
RprY-LacZ production in *E.coli*. A. *E. coli* strain MG1655 (ΔlacZ) carrying the *rprY-lacZ* fusion was subcultured in LB broth with or without NaCl (LB0). β-Galactosidase was assayed after 2 hr growth at 37°C. LB0 was supplemented with 171 mM NaCl, 171 mM KCl or 350 mM sorbitol to measure their effects on *rprY-lacZ* production. The results are the mean of three independent experiments. B. The RprY-lacZ fusion strain was diluted in LB or LB0 and incubated for 2 hrs, followed by addition of LiCl (500 mM) to the culture grown in LB medium. β-Galactosidase production was measured over time from cells cultured in LB (control): black diamonds; LB0: black circles; and LB+LiCl (500 mM): black squares. The experiment was performed twice and a representative experiment is shown.

We constructed *lacZ* protein fusions to study expression of *rprY* in response to various stimuli. Although up-regulated expression of a response regulator is not necessarily required under inducing conditions, increased expression due to a stimulus may be an indicator of activation. In *E. coli* K12 (the intermediate cloning host) we did observe significant expression (>500 Miller units) of the *rprY-lacZ* fusion in normal LB, suggesting that the *rprY* promoter was active in *E. coli* (Fig. 1A). This view was also supported by an earlier study in which *rprYX* genes from *B. fragilis* conferred tetracycline resistance and decreased *ompF* expression in *E. coli* [8].

Since RprY belongs to EnvZ-OmpR family of regulators (18) and our previous results indicated that RprY regulates sodium-dependent channels [9], we determined the effect of NaCl on RprY expression. In this experiment, normal LB medium (LB0+ NaCl) contains separately added 171 mM NaCl. As shown in Fig. 1A, omission of NaCl (LB0) caused a three- to six-fold increase in expression of the LacZ fusion, although there was an approximate three-fold decrease in growth of the strain (not shown). However, the increase in LacZ expression still occurred when sorbitol at equivalent osmolarity (500 mM) was substituted for NaCl (i.e., LB0+ sorbitol) which did not compromise growth (not shown), indicating that growth rate did not affect expression of the lacZ fusion. On the other hand, substitution with KCl (LB0+KCl) conferred lower expression similar to that in normal medium. (Not shown is that as much as 400 mM additional NaCl to normal medium had no effect).

The *E.coli rprY- lacZ* fusion strain was grown in complete and NaCl-depleted LB and in complete LB containing lithium chloride (LiCl). As a substrate analogue of Na, Li uses many of the same permeases for uptake, therefore when Li**^+^** is present in excess bacterial cells use it instead of Na**^+^**, leading to disruption of the normal sodium cycle [21]. *RprY- lacZ* fusion expression was assayed at different times during growth in three media: normal LB with and without Na^+^, and normal LB with LiCl (500 mM). Expression of *rprY-lacZ* was elevated for up to 150 mins in Na^+^ -depleted broth before declining (Fig. 1B). The data indicated that the *rprY* promoter responded immediately to Na^+^ depletion and also suggested that after cells adapted to the condition *rprY* expression is no longer required. Addition of LiCl resulted in growth retardation of the strain (data not shown), however the expression of the *rprY-lacZ* fusion increased up to 90 mins after addition of LiCl suggesting that the uptake of Li^+^ caused Na^+^ depletion (Fig. 1B). In summary, these experiments indicated that depletion of Na^+^ activated expression of the *rprY* promoter in *E. coli* and expression of *rprY* was affected by monovalent cation concentration and not osmotic stress.

### Sodium Depletion Prevents Growth of a *P. gingivalis rprY* Mutant

**Figure 2:**
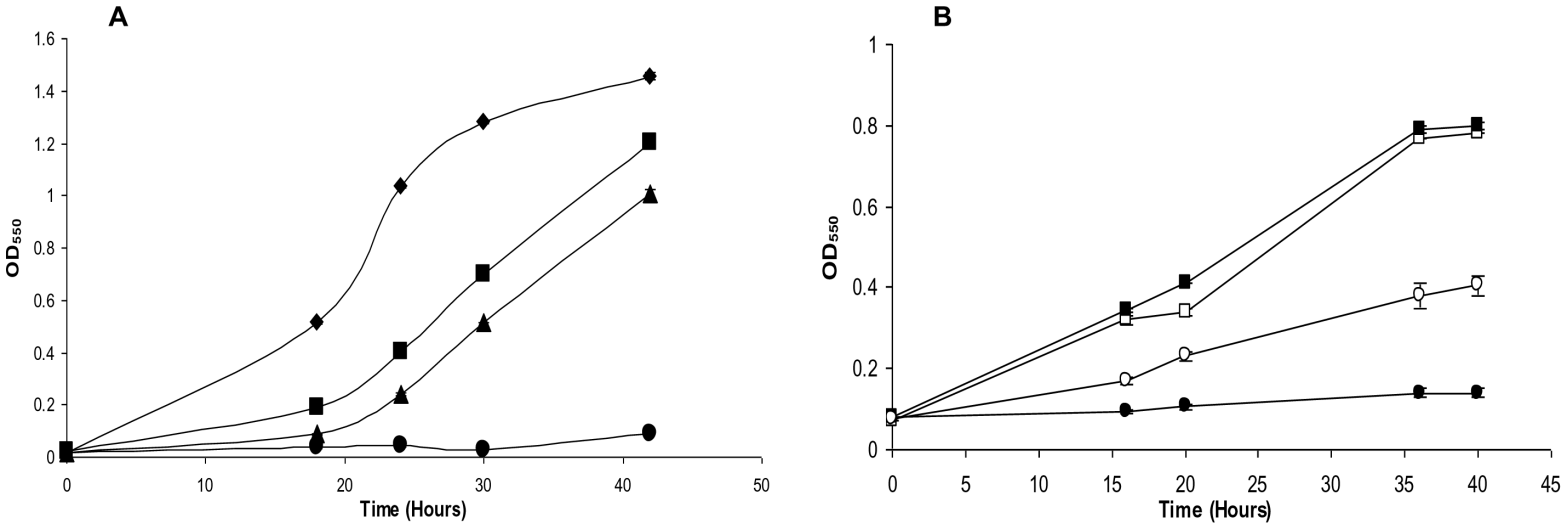
Growth of *P. gingivalis* strains in the absence of NaCl. Overnight cultures of *P. gingivalis* parent, *rprY* mutant (A), and complemented *rprY* mutant strains (B) were diluted 1∶40 (OD550 ∼0.02) in pre-reduced TSB media with or without NaCl and grown anerobically at 37°C. Parent strain with NaCl: black diamonds; *rprY* mutant with NaCl: black squares; parent without NaCl: black triangles; *rprY* mutant without NaCl: black circles; complemented *rprY* mutant with NaCl: open squares; complemented mutant without NaCl: open circles. The average of two independent experiments is shown. The experiment was repeated at least three times.

It was clear from our experiments with *E. coli* that RprY responds to Na^+^ depletion and thus may be involved in sodium-dependent metabolism. Next, we determined whether RprY played a role in the response of *P. gingivalis* to Na^+^ depletion, starting with the effect on growth. Overnight cultures of *P. gingivalis* ATCC 33277 parent and *rprY* mutant strains were grown in normal TSB medium, then diluted 1∶40 into (pre-reduced) normal or Na^+^-depleted TSB and incubated under anaerobic conditions. The *rprY* mutant grew slower than the parent even in complete medium, but in the absence of Na^+^ did not grow for up to 42 hours (Fig. 2A). These data indicated that the *rprY* mutant was hypersensitive to Na^+^ depletion and was unable to adapt to this growth condition. Complementation of the *rprY* mutation with the wild type gene on a plasmid partially restored growth of the mutant in the absence of Na^+^ (Fig. 2 B), indicating that RprY function was responsive to sodium.

### Sodium Depletion Induces an Oxidative Stress Response in *P.gingivalis*

**Figure 3:**
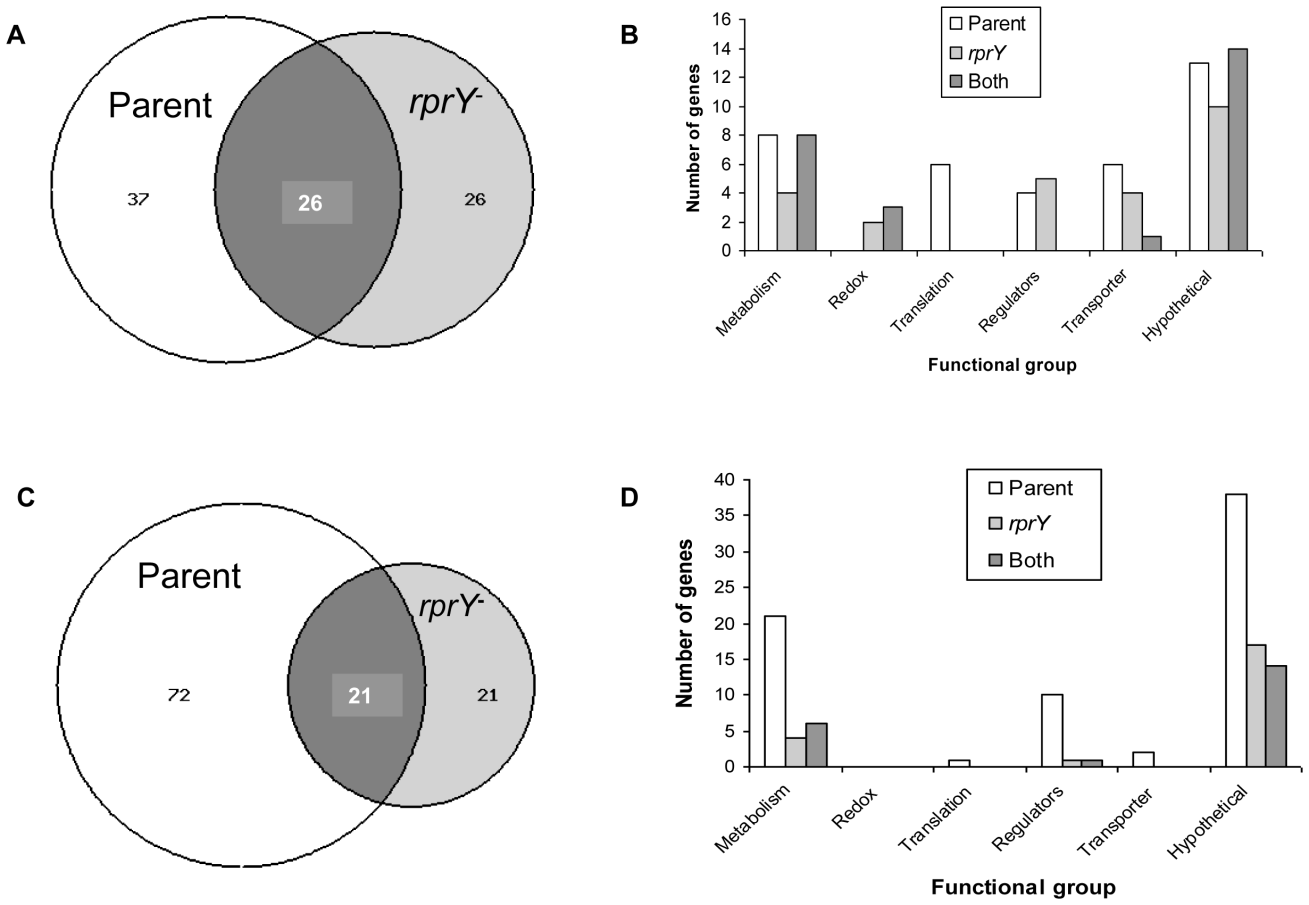
Gene expression analysis of parent and *rprY* mutant strains under Na^+^ depleted conditions. A and C. Venn diagrams showing functional categories and numbers of genes up-(A) or down-regulated (C), respectively, in the absence of Na^+^. B and D. Functional categories and numbers of genes up-(B) or down-regulated (D), respectively, in the absence of Na^+^. Open columns represent the number of genes up-regulated in the parent only, grey columns show the number up-regulated in the mutant only, and black columns the number of up-related genes in common.

We used comparative gene expression profiling to analyze further the role of RprY in the physiology of *P. gingivalis*. Because the *rprY* mutant cannot grow in Na^+^-depleted TSB we compared expression profiles after parent and mutant strains were grown to mid-logarithimic phase in complete TSB, then centrifuged and resuspended in modified TSB with or without NaCl, and incubated for 2 hrs anaerobically. Induction of *rprY-lacZ* in *E. coli* was rapid; therefore, to capture adequately the RprY response in slower-growing *P. gingivalis* we previously established by QRT-PCR that RprY was expressed in the parent strain after 2 hrs in the absence Na^+^ (data not shown). Using a conservative cut-off of 1.9-fold expression in either parent or mutant strains, a total of sixty-three genes were up-regulated in response to Na^+^ depletion (Na**^−/^**Na**^+^**) in the parent strain (Fig. 3A). The majority of these genes coded for hypothetical proteins, as shown in Fig. 3B. The next highest class of up-regulated genes were those associated with metabolism. A total of fifty-two genes were up-regulated in the *rprY* mutant upon Na^+^ depletion, again with the highest number coding for hypotheticals, followed by those involved in gene regulation or transport functions. Parent and mutant strains had twenty-six up-regulated genes in common. Of genes that responded to redox, at least two were uniquely up-regulated in the mutant while the rest were also upregulated in the parent. Regulators induced by Na^+^ depletion were either parent- or mutant-specific. Many more genes were down-regulated in the parent in response to Na^+^ depletion, i.e. ninety-three compared to forty-two in the mutant, and of these twenty-one genes were common to both strains (Fig. 3C and D). Again, the largest numbers of genes down-regulated in response to Na^+^ stress were hypotheticals and those involved in metabolic functions. Interestingly, few genes associated with redox, translation, and transport functions were down-regulated in either strain, but expression of more regulators was repressed in the parent including PG0020 (MarR family transcriptional regulator), PG0245 (universal stress protein family), PG0928 (response regulator), and PG0985 (ECF, RNA polymerase sigma-70 factor). The complete microarray data can be viewed at the Bioinformatics Resource for Oral Pathogens (www.brop.org).

In both parent and mutant strains Na^+^ depletion lead to significant upregulation of genes previously identified as responsive to oxidative stress in *P. gingivalis* [22], [23]. As shown in Table 2, these included formate-tetrahydrofolate ligase (PG1321), formate-nitrite transporter (PG0209), thiol peroxidase (PG1729), ferritin (PG1286) and thioredoxin (PG0034). In the *rprY* mutant upregulation of these genes was more robust and included the induction of additional oxidative stress response genes e.g. Fe-Mn superoxide dismutase (PG1545), thioredoxin family protein (PG0275), and tetrapyrrole methylase family protein (PG0433), and to lesser levels alkyl peroxidase reductase C (*ahpC*, PG0618), nitrite reductase-related protein (PG2213), a putative membrane protein (PG1868), and ATP:cobalamine adenosyltransferase (PG1124).

**Table 2:** Genes involved in the oxidative stress response that are differentially expressed under Na^+^ depleted conditions.

		Parent ATCC 33277−/+Na^+^			*rprY* mutant −/+Na^+^		
Locus^a^	Common name	Fold exp^b^	P val^c^	Repeats	Fold exp	P val	Repeats
PG0034	Thioredoxin	2.95	0.0004	12	3.25	0.0001	12
PG0046	Phosphatidate cytidylyltransferase	2.21	0.0028	12	2.25	0.0001	12
PG0047	Cell division protein FtsH, putative	2.04	0.0026	12	2.12	0.0016	12
PG0095	DNA mismatch repair protein MutS	0.65	0.0806	8	1.91	0.0041	8
PG0209	Formate-nitrite transporter	3.71	0.0004	12	3.39	0.0241	11
PG0258	ABC transporter, ATP-binding protein	1.78	0.0028	12	3.94	0.0000	12
PG0275	Thioredoxin family protein	1.71	0.0049	12	4.09	0.0000	12
PG0432	NOL1-NOP2-sun family protein	2.82	0.0517	12	3.58	0.0001	12
PG0433	Tetrapyrrole methylase family protein	2.88	0.0165	12	7.43	0.0001	8
PG0618	Alkyl hydroperoxide reductase, C subunit	1.17	0.1644	12	1.85	0.0119	12
PG0889	Peptidase, M24 family	2.35	0.0004	12	2.94	0.0002	12
PG0890	Alkaline phosphatase, putative	2.37	0.0002	12	3.46	0.0000	12
PG1043	Ferrous iron transport protein B	0.96	0.7002	7	2.01	0.0501	9
PG1044	Iron dependent repressor, putative	1.50	0.2211	12	3.27	0.0067	8
PG1088	Acetyltransferase, GNAT family	2.14	0.0001	12	0.94	0.5204	12
PG1089	DNA-binding response regulator RprY	1.58	0.0001	12	2.05^d^	0.0000	12
PG1124	ATP:cob(I)alamin adenosyltransferase, putative	1.12	0.4240	12	2.02	0.0000	12
PG1134	Thioredoxin reductase	1.27	0.0248	12	1.93	0.0075	12
PG1190	Glycerate dehydrogenase	1.56	0.1968	12	4.20	0.0000	10
PG1286	Ferritin	2.03	0.0005	12	4.00	0.0000	12
PG1321	Formate–tetrahydrofolate ligase	4.79	0.0001	12	6.78	0.0000	12
PG1545	Superoxide dismutase, Fe-Mn	1.96	0.0069	12	4.44	0.0001	12
PG1640	DNA-damage-inducible protein F	1.60	0.0011	12	2.16	0.0001	10
PG1642	Cation-transporting ATPase, authentic frameshift	1.88	0.0000	12	3.50	0.0000	12
PG1729	Thiol peroxidase	3.03	0.0000	12	3.68	0.0000	12
PG1841	Conserved hypothetical protein	3.20	0.0006	12	4.02	0.0005	12
PG1868	Membrane protein, putative	1.50	0.4660	10	3.83	0.0013	12
PG2008	TonB-dependent receptor, putative	1.03	0.8227	5	3.11	0.0620	8
PG2213	Nitrite reductase-related protein	1.54	0.0004	12	2.26	0.0005	12

a Loci numbers are those from the annotation of the genome of strain W83 and the microarrays.

b Fold expression.

c *P* values were calculated using LIMMA.

d The microarray PCR amplicon for *rprY* is upstream from the erythromycin resistance cassette that was used to generate the *rprY* mutation, thus an expression signal was still detectable.

**Figure 4:**
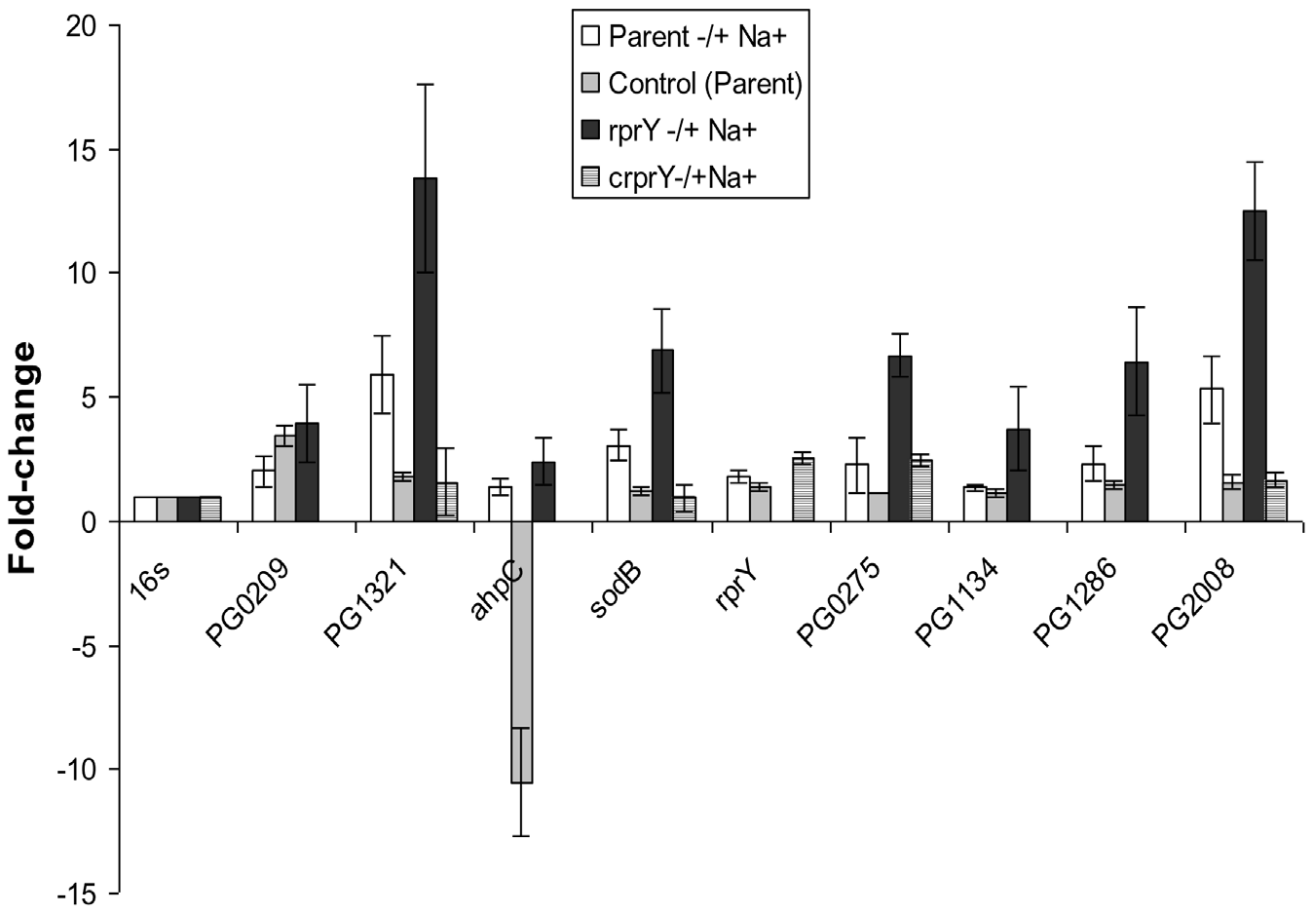
Confirmation of oxidative stress gene expression by QRT-PCR. The expression of several oxidative stress genes that were differentially regulated in Na^+^ depleted versus Na^+^ replete conditions was quantified by QRT-PCR. Positive fold values refer to increased, and negative values refer to decreased expression under Na^+^ depleted conditions. To verify that culture handling did not induce an oxidative stress response, control fold-expression changes (grey columns) were calculated by comparing gene expression in parent cultures processed immediately after growth with that in cultures after centrifugation and resuspension in pre-reduced normal TSB and anaerobic incubation (2 hrs). PG209: formate nitrate transporter; PG1321:formate tetrahydrofolate reductase; PG0275: thioredoxin family protein; PG1134: thioredoxin reductase; PG1286: ferritin; PG2008: putative TonB-dependent receptor.

Preliminary QRT-PCR control experiments ruled out the possibility that brief handling of cultures outside the anaerobic chamber induced this response. Differential regulation of several of these genes was confirmed by qRT-PCR (Fig. 4) in which the fold- changes of the genes tested was higher than seen by microarray, however, the trends of differential regulation were consistent with the microarray data. In addition, for the majority of transcripts assayed, complementation of the *rprY* mutant with the wild type gene restored expression to parental levels.

Overall, these results indicate that Na^+^ depletion induced an oxidative stress response. The “super” oxidative stress response in the *rprY* mutant may result from increased superoxide production in the mutant upon Na^+^ depletion. On the other hand, the data are also consistent with a model in which RprY directly or indirectly represses expression of several stress response genes.

To further verify that Na^+^ depletion induces an oxidative stress response of *P. gingivalis,* we carried out comparative analyses of small metabolites formed in parent and *rprY* mutant cells grown (2 hr) in the presence and absence of Na^+^. The data was analyzed in several ways including differences between parent and mutants strains in normal medium; in Na^+^-depleted medium; and metabolites that responded to Na^+^ stress in parent or mutant. The *p*-values obtained from Two-Way ANOVA statistical analyses indicated whether differences were due to the effect of the *rprY* mutation or to Na^+^ stress or both. Accordingly, as seen in Table 3, certain significant effects appeared to relate to the sodium status, e.g., in Na^+^ stress, higher methionine sulfoxide, lower ketosphingosine, and more of certain dipeptides. Other effects appeared more related to RprY function than to Na^+^, e.g., in the mutant, higher undecanoate and lower thiamine. While it is difficult to make close connections of these changes with physiology (see Discussion), we here observe only that this initial analysis of a large number of metabolites from biosynthesis, catabolism and central metabolism reveals perturbations in relatively few of them in the four situations examined.

**Table 3:** Small metabolite analysis.

Class	Compound	Relative Values				Two-Way ANOVA	
		Parent (−) Na^+^ Parent (+) Na^+^	*rprY* (+) Na^+^ Parent (+) Na^+^	*rprY* (−) Na^+^ *rprY* (+) Na^+^	*rprY* (−) Na^+^ Parent (−) Na^+^	Genotype Main Effect^a^	Treatment Main Effect^a^
						*p*-Value	*p*-Value
Lysine metabolism	Diaminopimelate	0.90	3.88	0.32	1.37	**0.0022**	**0.0112**
Methionine metabolism	Methionine sulfoxide	2.98	0.91	3.12	0.96	0.7805	**<0.001**
Dipeptides	Alanylglutamate	3.93	0.75	3.79	0.72	**0.0538**	**<0.001**
	Alanylleucine	1.99	0.62	3.04	0.94	0.5429	**0.0141**
Monoacylglycerol	1-Myristoylglycerol	0.40	1.50	0.13	0.50	0.9050	**0.0078**
	1-Palmitoylglycerol	0.75	4.44	0.11	0.65	0.1367	**0.0061**
Medium chain fatty acids	Undecanoate (11∶0)	1.61	10.64	0.94	6.20	**<0.001**	0.3274
Sphingolipids	3-Ketosphinganine	0.18	6.04	0.15	5.09	**0.0020**	**0.0050**
Thiamine metabolism	Thiamin (Vitamin B1)	1.06	0.18	1.40	0.24	**0.0971**	0.6976

a Specific effects, gene or Na^+^ stress are labeled in bold.

**Figure 5:**
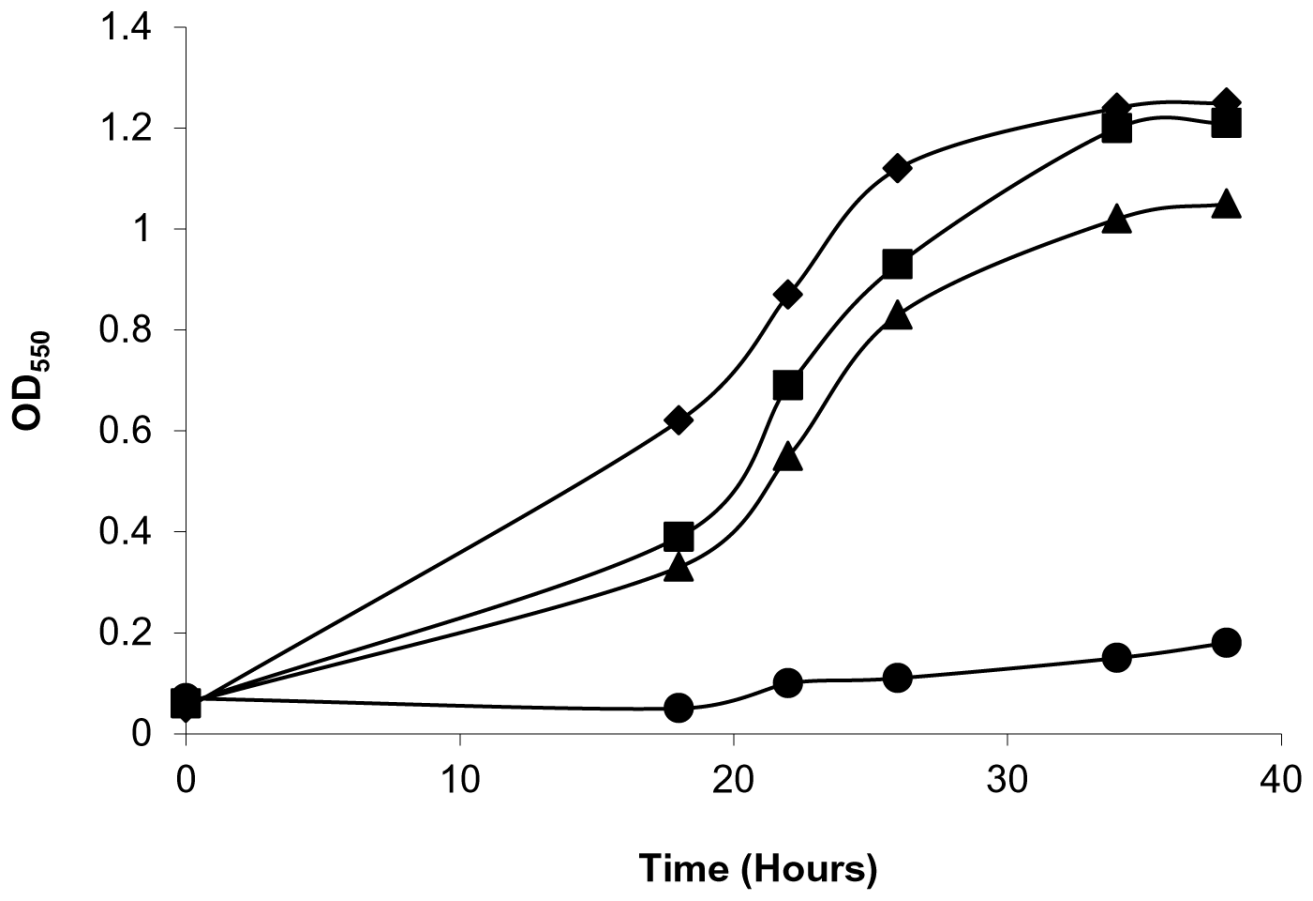
Growth of a *P. gingivalis oxyR* mutant in the absence of NaCl Overnight cultures of *P. gingivalis* parent and *oxyR* mutant strains were subcultured in medium with and without NaCl and grown anerobically at 37°C. Parent strain with NaCl: black diamonds; *oxyR* mutant with NaCl: black squares; parent without NaCl: black triangles; *oxyR* mutant without NaCl: black circles. A representative of at least three independent experiments is shown.

Finally, we compared growth of a *P. gingivalis oxyR* mutant with that of the parent strain in Na^+^-depleted and normal media. As observed previously, the parent strain was able to grow in the Na^+^-depleted medium while the *oxyR* mutant could not (Fig. 5), similar to the *rprY* mutant (Fig. 2). Since OxyR is a master regulator of genes involved in oxidative stress in Gram-negative bacteria [18], we infer that Na^+^ depletion causes oxidative stress in the *P. gingivalis* cells.

### RprY Directly Regulates Heat Shock Chaperones

**Figure 6:**
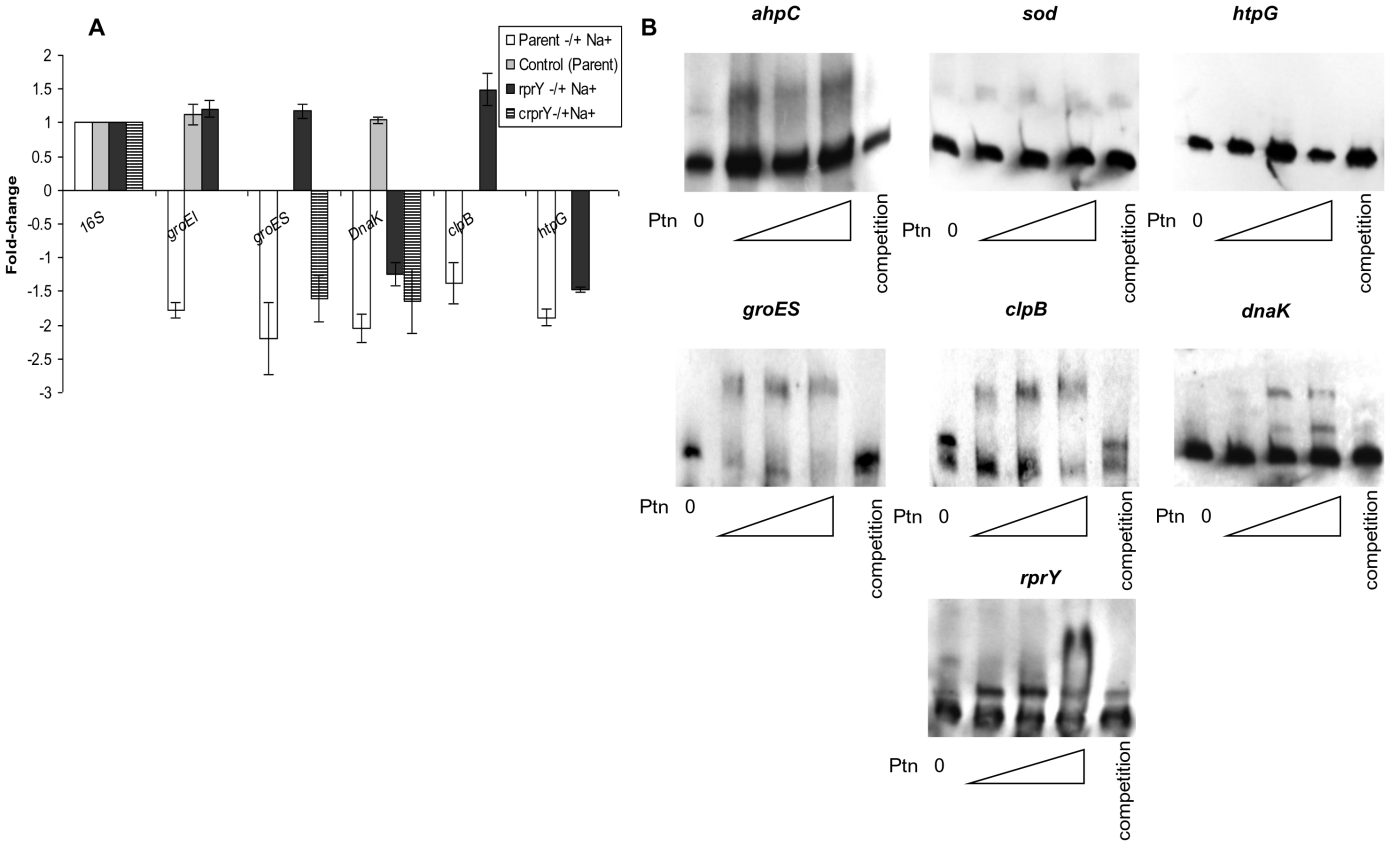
RprY and expression and regulation of chaperones. A. Expression of a subset of heat shock proteins was quantified by qRT-RTPCR. Positive- fold values refer to increased and negative values refer to decreased expression under Na^+^ depleted conditions. B. EMSA showing direct binding of RprY protein (0, 8.8, 17.6, and 35.2 pmoles) to the promoters of *clpB*, *groES, dnaK,* and *rprY*. The promoters of *sod* and *ahpC* were used as a negative and positive controls, respectively.

Heat shock chaperones and stress response proteases are expressed during adverse conditions to protect cells by ensuring correct protein folding and removal of denatured proteins. We observed significant downregulation of chaperones (Dnak, GroEL, GroES and HtpG) and ClpB and ClpC proteases due to Na^+^-depletion in the parent strain (Table 4), however, similar responses were not detected for *groESL* and *clpB* in the *rprY* mutant suggesting an inability to regulate expression of these defence proteins. This differential regulation was confirmed by qPCR with a subset of heat shock genes (Fig. 6A). Complementation of the *rprY* mutation restored expression of *groES* and *dnaK* transcripts to parental levels. Preparation of cells prior to incubation with and without Na^+^ did not affect expression of these genes (control).

**Table 4:** Differential regulation of heat shock and chaperone genes under Na^+^ depleted conditions.

		Parent ATCC 33277−/+Na^+^			*rprY* mutant −/+Na^+^		
Locus	Common name	Fold exp	*P-* val	Repeats	Fold exp	*P-* val	Repeats
PG0010	ATP-dependent Clp protease, ATP-binding subunit ClpC	1.20	0.6093	12	2.44	0.0007	12
PG0045	Heat shock protein HtpG	0.52	0.3206	11	1.01	0.9714	11
PG0245	Universal stress protein family	0.45	0.0002	5	0.82	0.0959	10
PG0520	Chaperonin, 60 kDa	0.55	0.2643	12	0.90	0.2939	12
PG0521	Chaperonin, 10 kDa	0.34	0.0719	10	0.90	0.1981	8
PG1118	ClpB protein	0.22	0.0000	5	1.44	0.2589	12
PG1208	DnaK protein	0.39	0.2538	8	1.04	0.8673	12
PG1775	GrpE protein	0.18	0.0000	5	0.66	0.0031	9

These data suggested that RprY may play a role in regulating the heat shock response in *P. gingivalis*. We performed electrophoretic mobility shift assays (EMSA) to determine whether the regulation was due to direct binding of RprY to the promoter regions of the genes in question or an indirect effect because of RprY interaction with another regulator. DNA probes were generated by amplification of the 5′ intergenic region upstream of the ORFs. DIG-labeled probes were incubated with increasing amount of phosphorylated rRprY protein (∼0, 8.8, 17.6 and 35.2 pmol). Protein binding specificity was determined by adding 50–100X excess unlabeled probe to the reaction mixes. In these experiments the *ahpC* and *sod* promoters were used as positive and negative controls, respectively, as we previously showed that *ahpC*, but not *sod*, was a target of RprY [9]. By EMSA we showed that RprY bound to the promoter regions of *groES, clpB* and *dnaK*, but did not bind to the promoter region of *htpG* (Fig. 6B).

The down regulation of chaperones in response to sodium depletion in the parent strain and the lack thereof in the *rprY* mutant suggested that RprY may be a negative regulator (repressor), and prompted an investigation of whether RprY was self-regulated. By EMSA we showed rRprY bound to its own promoter, lending support to the notion that RprY function was autoregulated, and possibly in response to the same stimulus of Na^+^ depletion.

## Discussion

*Porphyromonas gingivalis* is an opportunistic pathogen found in relatively low numbers in the healthy gingival crevice. During infection, bacteria are exposed to variable oxygen tension, ions, and proteins from saliva, serum and blood, and have evolved mechanisms to sense and respond to environmental changes and ensure survival. Conditions during periodontal infection such as low redox and the presence of host proteins and peptides favour growth of *P. ginigvalis*. As an obligate anaerobe, it is not surprising that the organism has developed redundant pathways to sense and respond to oxygen tension [24]. Previously, we identified gene targets of response regulator RprY by DNA-binding assays and comparative transcriptional profiling of parent and mutants strains grown under anaerobic conditions in complete medium (Na^+^ replete). Among the targets identified were NQR, the primary sodium pump; alkyl hydroperoxidase; ruberythrin; a ferrous iron transporter; and an iron -sulfur assembly system. From these results we hypothesized that RprY might play a role in the oxidative stress response, acting as both an activator and repressor [9].

Although there is high homology between regulator RprY from *P. gingivalis* and oral and enteric Bacteroidetes, the RprX sensor from these genera shares only weak homology with *P. gingivalis* histidine kinases, specifically at their C-termini. RprY does not contain transmembrane domains and must rely on a partner sensor kinase for perception of environmental cues and activation signal relay. Candidates for this function are still being sought, but include an orphan sensor kinase (PG0052) and possibly those paired with other response regulators. To circumvent the problem of the unknown sensor kinase, we used a reporter protein fusion approach to identify conditions that activate RprY using a construct in which the *rprY* promoter region and 41 bp of N-terminal coding nucleotides (13 amino acids) were fused to l*acZ*. Protein fusion expression was detected in *E. coli* implying a sensing and phosphorelay system that could activate the *P. gingivalis rprY* promoter. Interestingly, RprY expression in *E. coli* was dramatically induced in medium without NaCl and we demonstrated that specifically depletion of Na^+^ induced expression (Fig. 1). In *P. gingivalis*, omission of NaCl from culture media lead to a significant lag in the growth of the parent strain compared to growth in complete medium. The lag observed when the *rprY* mutant was grown in complete medium was also followed by growth recovery, however, the growth was not restored in the absence of NaCl. These differences in growth in Na**^+^** -replete and -depleted media may result from disruption of Na**^+^**-dependent metabolic processes such as protein folding, transport of growth substrates, and energy pathways. However, an *oxyR* mutant of *P. gingivalis* showed a similar growth phenotype in Na^+^ -depleted medium (Fig. 5), suggesting that condition induced an oxidative stress response that could not be controlled in the mutant.

In the present study comparative transcription profiling was carried out under more specific conditions, i.e. 2 hr in medium with and without Na^+^ (Table 2). Under Na^+^-depleted conditions both parent and mutant strains appeared to undergo oxidative stress as evidenced by up-regulation of genes known to be involved in the response [9], [22], [23], [25]. In most cases the relative levels of expression were greater in the *rprY* mutant than in the parent indicating that the regulator controls expression of genes important for growth and survival under these conditions. The preparation (centrifugation) of cells for Na^+^ stress experiments did not itself induce oxidative stress, consistent with the view that Na^+^ depletion was the cause. That the oxidative stress response was greater in the *rprY* strain than in the parent, suggests either that the mutant produced more oxidation products or the response could not be controlled adequately in the absence of RprY, which possibly functions as a repressor.

The proposal that Na**^+^** depletion induced an oxidative stress response in *P. gingivalis* was supported by analyzing the production of small molecule metabolites (Table 3). Levels of DAP increased almost four-fold in the *rprY* mutant under normal growth conditions and decreased in the Na^+^-depleted condition. Statistically, this result indicated a gene-specific effect rather than treatment, suggesting that an RprY-regulated function is limiting under Na^+^ stress. An important component of the bacterial cell wall, DAP can play a role in protection against unfavorable conditions, e.g. higher levels of DAP have been observed in cells grown in biocide -supplemented media [26]. Accumulation of DAP was possibly associated with the long lag growth phase (10 to 20 hr) of the mutant in normal medium. Eventually the mutant appeared to adapt and grow in normal medium, while in the absence of Na^+^ oxidative conditions preclude recovery.

In Gram-negative bacteria, a response to nutrient limitation is production of polyhydroxyalkanoates, storage compounds that are produced in unbalanced metabolic conditions [27], [28]. In the present study, *rprY* mutant cells contained high levels of undecanoate, a subunit of a polyhydroxyalkanoate, which appeared to be a gene effect. Interestingly, polyhydroxyalkanoate production decreased the oxidative stress response in a psychrophilic Pseudomonas species by modulating the levels of reducing equivalents [29]. Thiamine levels were approximately five-fold lower in the mutant strain than in the parent, another effect of the *rprY* mutation. A link between thiamine and oxidative stress was provided by the observation that thiamine supplementation restored the growth of *Salmonella enterica* on paraquat, a superoxide-generator, the interpretation being that oxidative stress directly caused the thiamine requirement [30]. Our data would suggest that low levels of thiamine in the *rprY* mutant result from oxidative stress induced by the mutation, even in normal media.

It has been hypothesized that, as in eukaryotes, sphingolipids, may be involved in sensing and signalling in response to stress in *Bacteriodes fragilis* [31]., Stationary phase cells of *B. fragilis* either chemically or nutritionally deprived of sphingolipids were less able to survive oxidative stress and DNA crosslinking, leading to the proposal that sphingolipid-rich domains in the periplasmic membrane may transmit environmental information by signalling relays to transcription factors that regulate appropriate responses [31]. Other Bacteroidetes, including *Porphyromona*s, also contain sphingolipids [32]. 3-Ketosphinganine is a direct presursor of sphinganine, and we observed that upon Na**^+^** depletion, both parent and *rprY* mutants cells contained significantly lower amounts of this intermediate (approximately six-fold reductions). The statistics suggested that this was a treatment effect, the absence of Na^+^ having induced stress, however, we do not yet know whether the low levels of 3-ketosphinganine are a direct result of Na^+^ depletion.

During Na^+^ stress both parent and mutant cells contained higher levels of intracellular methionine sulfoxide, attributable to the treatment rather than a mutation. Methionine is modified to methionine sulfoxide by reactive oxygen species and to repair oxidative damage the sulfoxide is converted back to methionine by methionine sulfoxide reductases. Interestingly, in the response of *B. fragilis* to air exposure methionine sulphoxide reductase was induced more than ten-fold [33], however, there were no significant changes in the expression of this gene in *P. gingivalis* strains under Na^+^ stress (data not shown). As an asaccharolytic organism, *P. gingivalis* preferentially uses dipeptides as carbon, nitrogen and energy sources. The metabolite data indicated that in addition to the preferred aspartate and glutamate containing peptides, Na^+^ stressed cells showed an increased ability to use diverse peptides, notably alanyl peptides with leucine and glutamate. Whether this expansion of growth substrates is an adaptation that offsets growth limiting conditions is as yet unknown.

The *oxyR* mutant also failed to grow in Na^+^-depleted medium, implying that this condition mimics oxidative stress (Fig. 5). Approximately half the genes up regulated in both ATCC 33277 parent and *rprY* mutant strains under Na^+^ stress were reported to be associated with the OxyR regulon [18]. It was demonstrated that expression of several of these genes was maximal during normal anaerobic growth and were downregulated in an *oxyR* mutant grown under the same conditions, consistent with OxyR was a positive regulator of these genes [18]. Our data show that under Na^+^ stress expression of these genes was higher in the mutant consistent with RprY being a repressor. A possible scenario is that under anaerobic conditions OxyR constitutively activates oxidative stress genes and RprY, acting as a repressor, fine- tunes the expression of a subset of these to maintain a balance that prevents a superoxidative stress response. Sodium depletion disrupts the balance triggering induction of RprY in order to restore homeostasis and eventual growth as in the parent strain. However, in the absence of RprY a superoxidative stress response ensues from which the mutant cannot recover and grow.

Transcriptome comparisons between parent and *rprY* mutant strains in Na**^+^**-depleted conditions also revealed differential expression of certain chaperones and heat shock proteins that were down- regulated in the parent but not in the *rprY* mutant (Table 4); we confirmed these differences in a subset of genes by QRT-PCR (Fig. 6). Molecular chaperones are important for maintaining the quality of the proteins expressed in the cells through stabilization and correct folding primarily supported by trigger factor followed by the DnaKJ and GroESL chaperone systems [34], [35]. Certain chaperones also play a role in disaggregation and refolding of misfolded proteins, e.g. protein aggregation induces sets of chaperones (DnaKJ, GroESL, ClpB, HtpG etc.) and proteases (ClpXP, Lon etc), collectively termed heat shock proteins (HSP).

By EMSA we established that RprY binds to the promoter regions of *groES, dnaK* and *clpB*. We reasoned that Na**^+^** -depletion caused oxidation of metabolites and proteins, and induction of RprY. Parent cells mount a stress response that included activation of OxyR regulated genes, e.g. superoxide dismutase [25] and repression of RprY–regulated genes, e.g. *ahpCF* [9] as well as heat shock proteins and chaperones to protect cells from protein oxidation and misfolding. The transcription data suggests that RprY acts as a repressor of *dnaK*, *groESL* and *clpB* which may help the parent adapt to Na^+^ stress. In the mutant lacking RprY, *groESL, dnaK* and *clpB* expression cannot be repressed which may contribute to the growth defect upon Na^+^-depletion. The expression of heat shock proteins is tightly controlled by both positive and negative regulation [36], [37]. The expression of several of HSP chaperones is maintained at basal levels by mechanisms that include negative feedback loops [38] and blocking of promoters by CIRCE sequences and negative regulators [39]. Positive regulation is achieved by expression of alternate sigma factors [36]. Thus HSP expression is tightly coordinated with cell requirements during stress and non-stress conditions to ensure survival. Analysis of *Porphyromonas gingivalis* HSP promoter regions did not identify the classic CIRCE sequences required for negative regulation, leading to the assumption that *Porphyromonas* may utilize different control mechanisms.

RprY expression is induced under several environmental conditions including oxidative and nitrate stresses [40] suggesting an important role as a response regulator. The *rprY* mutant was unable to survive aerobic stress compared to the parent strain (data not shown). The interpretation of our data is that Na^+^ depletion leads to activation of RprY enabling it to bind to target promoters, including it’s own, and function as a repressor. The role of RprY in the response to Na^+^- depletion may be explained by two interlinked scenarios. First, absence of RprY leads to increased oxidative stress during Na^+^ depletion because of lower basal levels of the Na^+^-dependent ubiquinone oxidoreductase system (NQR) which RprY appears to regulate [9]. It has been proposed that NQR utilizes the sodium gradient it generates to produce energy for the CydAB system to convert oxidative radicals to water [41]; thus, lower levels of NQR may result in inefficient removal of these radicals. Second, mutation in *rprY* leads to loss of negative control of the optimal response by both oxidative stress and heat shock genes. As reported by Hase et al., many pathogens possess enzyme systems to generate sodium motive force in addition to or as alternatives to H+ motive force [21]. In *P. gingivalis* it was suggested that NQR may play a buffering role against Na^+^ released during periodontal infection and bleeding. However, our data imply that RprY is inactive in the presence of Na^+^, and the health conditions that lead to Na^+^ depletion in the oral cavity are, as yet, unknown.

## Ackowledgments
